# Differential Resistance Mechanisms to Glyphosate Result in Fitness Cost for *Lolium perenne* and *L. multiflorum*

**DOI:** 10.3389/fpls.2017.01796

**Published:** 2017-10-17

**Authors:** Pablo T. Fernández-Moreno, Ricardo Alcántara-de la Cruz, Reid J. Smeda, Rafael De Prado

**Affiliations:** ^1^Department of Agricultural Chemistry and Edaphology, University of Cordoba, Cordoba, Spain; ^2^Departamento de Entomologia BIOAGRO, Universidade Federal de Viçosa, Viçosa, Brazil; ^3^Division of Plant Sciences, University of Missouri, Columbia, MO, United States

**Keywords:** *Lolium* spp., resistance, glyphosate, mechanisms, fitness cost

## Abstract

Multiple mechanisms of resistance to glyphosate are exhibited by populations of *Lolium* spp. worldwide. Association of resistance with growth and reproductive fitness is an important predictor for long-term success of glyphosate-resistant (R) versus glyphosate-susceptible (S) biotypes. Numerous studies were conducted on R- and S-biotypes of Italian ryegrass (*Lolium multiflorum*) and perennial ryegrass (*L. perenne*) to characterize the underlying mechanism(s) of glyphosate resistance and associate this with growth and reproductive fitness. *L*. *perenne* expressed both altered uptake and translocation as well as a genetic change at 106-Pro to –Ser, This pattern for two resistance mechanisms is unique. *L. multiflorum* also exhibited altered uptake and translocation as well as duplication of EPSPS gene copies. Reduced plant biomass and height for R-versus S-biotypes of both species was evident over two growing seasons. This resulted in S- versus R- *L. multiflorum* producing up to 47 and 38% more seeds in 2014 and 2015, respectively. S- *L. perenne* produced up to 20 and 30% more seeds in 2014 and 2015, respectively. Both non-target site and target-site mechanisms of glyphosate resistance can render *Lolium* spp. at a competitive disadvantage. This has long-term implications for the success of glyphosate-resistant plants in the absence of selection pressure.

## Introduction

Over the past two decades, glyphosate (*N*-phosphonomethyl glycine) has been widely used world-wide for non-selective weed control in genetically modified crops, and for over four decades as a non-selective herbicide in crop and non-crop situations (Duke, 2017). Glyphosate (group G) inhibits the enzyme 5-enolpyruvlshikimate-3-phosphate synthase (EPSPS), which catalyzes the reaction of shikimate-3-phosphate (S3P) and phosphoenolpyruvate to form 5-enolpyruvyl-shikimate-3-phosphate (EPSP) (Maeda and Dudareva, 2012). Inhibition of EPSPS specifically results in accumulation of shikimate in sensitive plants and measurement of shikimate levels is a common method to ascertain resistance in selected species (Singh and Shaner, 1998). The result is prevention of biosynthesis of aromatic amino acids, which is highly lethal to sensitive plants (Sammons and Gaines, 2014). Metabolism in plants is limited; symptoms in treated plants are slow to develop, but plant death is evident within 20 days following treatment.

Long-term use of glyphosate has contributed to selection of over 37 weed species world-wide that exhibit resistance (Heap, 2017). Herbicide resistance is an evolutionary phenomenon, allowing resistant weeds exposed to the labeled dose of an herbicide to sustain growth with little or no symptomology (Fernández-Moreno et al., 2017a). Factors important in the development of herbicide-resistant species include strict dependence on one herbicide mode of action and continuous use (within and over years) of this herbicide mode of action until control failures are observed (Fernández-Moreno et al., 2017b).

Initial reports of glyphosate-resistant (R) *Lolium* species occurred in Australia in 1996 (Pratley et al., 1996; Powles et al., 1998). Currently, three *Lolium* species, *L. rigidum* Gaud., *L. perenne* L., and *L. multiflorum* Lam. exhibit glyphosate resistance, with populations scattered across 14 countries (Heap, 2017). Similar to species evolving resistance to other herbicides, R *Lolium* spp. resulted following exclusive use of glyphosate multiple times within and over growing seasons (Nandula et al., 2008; Bracamonte et al., 2016). The level of resistance in *L. rigidum* and *L. multiflorum* differs widely among populations, varying from 2- to 100-fold (Preston et al., 2009).

A plethora of mechanisms underlying resistance to glyphosate have evolved, and can be categorized into two groups: non-target site resistance (NTSR), and target site resistance (TSR) (Sammons and Gaines, 2014). Expression of NTSR mechanisms are common in many weed species, and essentially *in vivo* limit glyphosate from reaching the target enzyme in sensitive plants (Ghanizadeh and Harrington, 2017). This mechanism consists of reduced uptake and translocation, increased vacuolar sequestration, and metabolism to non-toxic compounds, resulting in decreased levels of glyphosate interacting with EPSPS (Ge et al., 2014; Fernández-Moreno et al., 2017b; Kleinman and Rubin, 2017). Levels of resistance conferred by the NTSR mechanisms are variable and unpredictable between plant species (Ghanizadeh et al., 2015). However, TSR mechanisms have been studied and reported more often than NTSR mechanisms.

Target site resistance mechanisms have been widely studied in plant species for many herbicide modes of action, and often underlie resistance. One TSR mechanism involves one or more mutations in the DNA encoding the target protein of the herbicide, which leads to changes in amino acids or conformational changes in protein folding, ultimately resulting in high levels of resistance (Sammons and Gaines, 2014; Fernandez et al., 2015; Yu et al., 2015; Alcántara-de la Cruz et al., 2016a). Sammons and Gaines (2014) summarized the single mutations in the Pro-106 position of the EPSPS gene that have resulted in resistance for a number of species. In addition, a double mutation was found in the Thr-102-Ile position as well as Pro-106-Ser position, conferring resistance in *Eleusine indica* (Chen et al., 2015; Yu et al., 2015). Alternatively, overexpression of the herbicide’s target protein confers a limited level of resistance. This mechanism is exemplified by amplification of genes encoding EPSPS protein (Gaines et al., 2010; Salas et al., 2012, 2015). Recently, glyphosate resistance involving two TSR mechanisms, Pro-106 mutation and EPSPS amplification, was discovered in a population of *E. indica* (Gherekhloo et al., 2017).

Evolved herbicide resistance and subsequent enrichment of resistant individuals by high selection pressure can result in large populations of resistant weeds (Vila-Aiub et al., 2009, 2011). In the continued presence of selection pressure, resistant weeds have an ecological advantage versus susceptible individuals of the same species. However, Vila-Aiub et al. (2014) theorized that in the absence of selection pressure, the cost of herbicide resistance may render plants at a competitive disadvantage.

The expression of reduced fitness associated with herbicide resistance varies with species and herbicide mode of action (Yanniccari et al., 2012a). Glyphosate resistance associated with fitness costs have periodically been identified (Yanniccari et al., 2016) for both NTSR and TSR mechanisms (Preston and Wakelin, 2008; Yu et al., 2015). Pedersen et al. (2007) reported that seed production of glyphosate-resistant (R) versus glyphosate-susceptible (S) *L. rigidum* was reduced, with TSR as the underlying mechanism of resistance. The objective of this research was to assess the level of glyphosate resistance in suspect resistant and susceptible biotypes of *L. multiflorum* and *L. perenne*. In addition, the mechanism(s) involved in both suspect *R*-glyphosate biotypes was assessed, as well as any associated growth and reproductive fitness cost.

## Materials and Methods

### Plant Material

Mature seeds of suspect R Italian ryegrass (*L. multiflorum*) biotype were collected from a vineyard located in Peso da Régua, Portugal. This vineyard was treated with 1080 g ae ha^-1^ of glyphosate (3 L ha^-1^) or higher for at least 15 consecutive years (Roundup^®^, 360 g ae L^-1^ as isopropylamine salt). Seeds of an S ryegrass biotype were collected from a nearby vineyard; glyphosate had never been used at that location. These biotypes will hereafter be termed R-Douro and S-Douro. In addition, seeds of R and S perennial ryegrass (*L. perenne*) biotypes were provided by the FITO^®^ seed company (Barcelona, Spain). These biotypes will hereafter be termed R-Golf and S-Golf (R-Golf is targeted for production on golf courses).

Seeds were germinated in petri dishes with moistened filter paper (distilled water). Germinating seedlings were transplanted into pots (7 × 7 × 6 cm) containing sand and peat (1:2 v/v) and placed in a growth chamber under the following conditions: 28/18°C (day/night); 16 h photoperiod, light intensity of 850 μmol m^-2^ s^-1^ photosynthetic photon flux density; and 80% relative humidity.

### Identification of *Lolium* Weed Species Using Molecular Markers

Following the methodology of Fernández-Moreno et al. (2017a), AFLP markers were used to characterize the genetic similarity of R and S biotypes for each *Lolium* species, enabling assessment if the populations belonged to different species. Plant material included two S biotypes (S-Golf and -Douro), and two R biotypes (R-Golf and -Douro). Twenty plants of each biotype were used for molecular analysis. Additionally, twelve reference S-plants (*L. multiflorum* and *L. perenne*) were included in the study.

DNA was extracted from the leaf tissue (50 mg) using the Speedtools DNA Extraction Plant kit (BIOTOOLS, Madrid, Spain). The quality and concentration of the DNA was evaluated by spectrophotometric analysis with light absorption at 260 and 280 nm. AFLP analysis was carried out using the fluorescent AFLP IRDye kit for Large Plant Genome Analysis (LI-COR Biosciences). Template preparation was performed following the protocol included in the kit, including digestions with EcoRI and MseI restriction enzymes (Invitrogen). Fernández-Moreno et al. (2017a) have described primers for selective amplification.

AFLP products were separated by polyacrylamide electrophoresis using an automated sequencer (LICOR 4300). Polymorphic AFLP markers and primers were identified and individuals were scored for the presence or absence of AFLP fragments using the computer package SAGAMX 2 GENERATION. UPGMA analysis was performed with AFLP marker data using the computer program NTSYSpc 2.2.

### Whole Plant Dose-Response

Seedlings at the 3–4 leaf growth stage were treated with glyphosate using a laboratory chamber (SBS-060 De Vries Manufacturing, Hollandale, MN, United States) equipped with 8002 flat fan nozzles and delivering 200 L ha^-1^ at 250 KPa. The isopropylamine salt formulation of glyphosate (Roundup Energy SL, 450 g ae L^-1^, Monsanto) was applied at: 0, 62.5, 125, 250, 500, 1000, 2000, and 4000 g ae ha^-1^. The experiment was arranged using nine replications and was repeated. Plant mortality and dry mass were evaluated 21 days after treatment (DAT). Plant dry mass was measured for above ground tissue after drying at 60°C for 72 h.

### Spray Retention Assay

At the 3–4 leaf growth stage, R- and S-plants of each biotype were sprayed with 300 g ha^-1^ of glyphosate and 100 mg L^-1^ Na-fluorescein using conditions as described above. Na-fluorescein was used as a labeling reagent to determine the amount of herbicide solution retained. Once the foliage had dried (30 min), shoot tissue was harvested and immersed in 50 mL of 5 mM NaOH for 30 s to remove spray solution. Fluorescein absorbance was determined using a spectrofluorometer (Hitachi F-2500, Tokyo, Japan) with excitation wavelength of 490 nm and absorbance at 510 nm. Plant tissue was dried for 48 h at 60°C and recorded. The experiment was arranged in a completely randomized design with four replications per biotype and repeated. Spray retention was expressed as mL spraying solution per gram dry matter.

### Shikimic Acid Accumulation

Fifty mg (4 mm leaf disks) were harvested from the youngest fully expanded leaf at the 3–4 tiller stage from 15 plants per biotype (Pedersen et al., 2007; Hanson et al., 2009). Five disks of fresh tissue were transferred to 2 mL Eppendorf tubes containing 1 mL of 1 mM NH_4_H_2_PO_4_ (pH 4.4). At this point, 1 μL of glyphosate was added to each tube resulting in the following concentrations: 0, 0.1, 0.5, 1, 5, 10, 50, 100, 200, 400, 500, 600, and 1000 μM. Tubes were incubated in a growth chamber for 24 h under the above conditions. After 24 h, tubes were stored at -20°C until further analysis. For analysis, tubes were thawed at 60°C for 30 min. Thereafter, 250 μL of 1.25 N HCL was added to each Eppendorf. Tubes were incubated at 60°C for 15 min. A 125 μL aliquot from each Eppendorf was pipetted into a new 2 mL Eppendorf, and 500 μL of periodic acid and sodium metaperiodate (0.25 % [wt/v] each) was added. After incubation at room temperature for 90 min, 500 μL of 0.6 N sodium hydroxide and 0.22 M sodium sulfite were added. Finally, liquid in the tubes was transferred to glass vials. Within 30 min, light absorption at 380 nm was measured in a spectrophotometer. For each glyphosate concentration and biotype, five replications were used and the experiment repeated.

### ^14^C-Glyphosate Uptake and Translocation

Experiments were carried out according to Fernández-Moreno et al. (2016). A solution of ^14^C-glyphosate (American Radiolabeled Chemicals, Inc., Saint Louis, MO, United States) was prepared by adding radiolabeled glyphosate to commercially formulated glyphosate, with the final specific activity of 0.834 kBq μL^-1^. This concentration corresponded to 300 g ae ha^-1^ and a volume of 200 L ha^-1^. When R- and S-biotype plants reached the 3–4 leaf growth stage, 1 μL (0.834 KBq plant^-1^) of solution was applied onto the adaxial surface of the second most mature leaf with a micropipette (LabMate Soft, HTL Lab Solutions, Warsaw, Poland).

Following 12, 24, 48, 72, and 96 h after treatment (HAT), the treated leaf was washed with 3 mL of water:acetone (1:1 v/v) solution to remove unabsorbed glyphosate. The rinsate was mixed with 2 mL of scintillation cocktail and radioactivity analyzed by liquid scintillation spectrometry (LSS) using a scintillation counter (Beckman LS 6500, Fullerton, CA, United States.). The remainder of the plant was carefully removed from the pot and roots gently washed with distilled water. Plant tissue was sectioned into treated leaf, remaining shoot tissue, and roots. Plant tissue was dried at 60°C for 96 h and combusted in a Packard Tri Carb 307 biological sample oxidizer (Packard Instruments, Meriden, CT, United States). Evolved ^14^CO_2_ was trapped and counted by LSS in an 18-mL mixture of Carbo-Sorb E and Permafluor E++ (1:1v/v) (Perkin-Elmer, Packard Bioscience BV). The quantity of radiolabeled glyphosate deposited on plant leaves was assessed by washing a plant leaf of each biotype immediately after treatment. The experiment was arranged in a completely randomized design with five replications per biotype and was repeated. The percentage of absorbed herbicide was expressed as: [kBq in combusted tissue/(kBq in combusted tissue + kBq in leaf washes)] × 100.

### ^14^C-Glyphosate Visualization

Distribution of ^14^C-glyphosate throughout the R and S plants was visualized using a phosphor imager (Cyclone, Perkin-Elmer, Waltham, MA, United States). Plants were grown, treated, and tissue collected in the same way as described for the uptake and translocation experiments. Following 96 HAT, roots of intact plants were rinsed to remove soil and treated leaves were rinsed to remove unabsorbed glyphosate. Following gentle blotting on absorbent tissue to remove water, plants were gently pressed and dried at room temperature. Dry tissue was placed adjacent to 25 × 12.5 cm phosphor storage film for 13 h and scanned for radiolabel distribution using a phosphor imager. The experiment was conducted once using three plants for each biotype.

### Metabolism Study

R- and S-biotypes were grown to the 3–4 leaf growth stage, and then treated with glyphosate at 300 g ha^-1^ as described in the dose response section. At 96 HAT, the methodology of Rojano-Delgado et al. (2010) was followed to determine glyphosate and the primary metabolites, aminomethylphosphonic acid (AMPA), glyoxylate, sarcosine, and formaldehyde. Quantification of glyphosate and metabolites was determined by reversed polarity capillary electrophoresis using a 3D Capillary Electrophoresis Agilent G1600A instrument equipped with a diode array detector (DAD) at a wavelength of 190–600 nm. For calibration of instrumentation, purchased standards of glyphosate, AMPA, sarcosine, formaldehyde, and glyoxylate were used. Preparation of treated leaf tissue for analysis was as follows: leaf tissue was washed with distilled water, frozen in liquid nitrogen, and stored at -40 °C until use. The aqueous background electrolyte consisted of 10 mM potassium phthalate, 0.5 mM hexadecyltrimethylammonium bromide, and 10% acetonitrile at pH 7.5. The calibration equations were established from non-treated plants and known concentrations of glyphosate and associated metabolites, which were determined from their peak areas in the electropherogram. The average value for the content of glyoxylate, which is naturally produced by plants, was subtracted from the mean content of each biotype. The experiment was arranged in a completely randomized design with five replications per biotype. Experiments with each biotype were repeated.

### EPSPS Enzyme Activity Assays

Samples of 5 g of leaf tissue (3–4 leaf growth stage) from each biotype were ground to a fine powder using a pestle and mortar. The methodology described by Sammons et al. (2007) was used for EPSPS extraction. The total content of protein in the extract was measured using a Kit for Protein Determination (Sigma–Aldrich, Madrid, Spain).

Specific EPSPS activity in plants from each biotype was estimated in the presence and absence (basal activity) of glyphosate. The EPSPS activity was determined using an EnzChek Phosphate Assay Kit (Invitrogen, Carlsbad, CA, United States). The glyphosate concentrations used were: 0, 0.1, 1, 10, 100, and 1000 μM. Five replications at each glyphosate concentration were used, and the experiment was repeated. EPSPS enzyme activity was expressed as percentage of enzyme activity in presence of glyphosate with respect to the control (without glyphosate). The EPSPS activity was calculated to determine the amount of phosphate (μmol) released per μg of total soluble protein (TSP) min^-1^.

### EPSPS Gene Sequence

Samples of young leaf tissue (∼100 mg) from 10 individual plants within each biotype were harvested and stored at -80°C. Total RNA was isolated from leaves using TRIzol reagent (Invitrogen, Carlsbad, CA, United States) according to the manufacturer’s instructions. RNA was purified with TURBO DNase (RNase-Free; Ambion, Warrington, United Kingdom) and stored at -80°C. Synthesis of cDNA utilized 2 μg of total RNA following an M-MLV (Moloney Murine Leukemia Virus) Reverse Transcriptase (Invitrogen, Carlsbad, CA, United States) in combination with oligo (dT)_12-18_ and random nonamers (Amersham Biosciences, Amersham, United Kingdom) according to the manufacturer’s instructions. To amplify the EPSPS gene, known primers (forward: 5′ AGCTGTAGTCGTTGGCTGTG 3′; reverse: 5′ GCCAAGAAATAGCTCGCACT3 ′) were used and PCR reactions were carried out using cDNA, 1.5 mM MgCl_2_, 0.2 mM dNTP, 0.2 μM of each primer, 1× buffer, and 0.625 units of a 100:1 enzyme mixture of non-proofreading (*Thermus thermophilus*) and proofreading (*Pyrococcus furiosus*) polymerases (BIOTOOLS, Madrid, Spain) in a final volume of 25 μL. All PCR reactions were in duplicate and cycling conditions included: 94°C, 3 min; 35 cycles at 94°C, 30 s; 55°C, 30 s; 72°C, 1 min; and a final extension cycle of 72°C, 10 min. An aliquot of the PCR product was loaded on a 1% agarose gel to assess amplification of the correct band. The remaining PCR product was purified using ExoSAP-IT^®^ (USB, Cleveland, OH, United States) as instructed. Ten purified PCR products per biotype were sequenced (STAB VIDA, Caparica, Portugal).

### EPSPS Gene Expression

The cDNA of five sequenced individuals, corresponding to the previous section, was used for qPCR analysis. The primer pair EPSPS F2 (5′-CTGATGGCTGCTCCTTTAGCTC-3′) and EPSPSR2 (5′-CCCAGCTATCAGAATGCTCTGC-3′) designed by Salas et al. (2012), was used. Moreover, the primers LpCCR1 F2 (5′-GATGTCGAACCAGAAGCTCCA-3′) and LpCCR1 R2 (5′-GCAGCTAGGGTTTCCTTGTCC-3′) (McInnes et al., 2002), corresponding to cinnamoyl-CoA reductase (CCR), gene expressed as a single copy gene in perennial ryegrass, were used as an internal standard to normalize the samples for differences in the amounts of cDNA. The qPCR conditions described by Salas et al. (2012) was followed, and the PCR-reactions conducted using an ABI Prism 7500 sequence detection system (Applied Biosystems, United States). EPSPS gene expression analyzes were performed according to Alcántara-de la Cruz et al. (2016b).

EPSPS expression level was calculated for each qPCR reaction. The PCR efficiency of each sample and the stability of the CCR were determined using geNorm software according to Vandesompele et al. (2002). Two-three technical replications per plant were arranged in a completely randomized design.

### Fitness Assessment

Progeny selection for both Douro and Golf biotypes was conducted by cloning plants following the methodology described by Yanniccari et al. (2012b). Plants were grown and vegetative clones of individual plants were propagated by tiller partition to obtain four ramets per plant. When ramets reached four leaves, plants were treated with 0, 360, 500, 720, 1000, and 2000 g ha^-1^ of glyphosate as described in the dose-response section. At 21 DAT, a visual assessment was made, with all plants not surviving 500 g ha^-1^ characterized as S, and those surviving 1000 g ha^-1^ or greater considered R. Cloned R- and S-plants were transferred to a greenhouse. Prior to flowering, cross pollination within R and S biotypes of each species was precluded by isolating plants in pollen-proof enclosures until maturity, and all seeds per plant were collected, enumerated, and stored at 4°C (seed did not exhibit a dormancy requirement). Cloning and selection was carried out in 2014 and again in 2015.

Seeds generated from the selection and isolation process described above were germinated in sand and peat medium and maintained in conditions described earlier. At 30, 60, 90, 120, and 150 days after planting (DAP), plant height (from soil level to flag sheet) and shoot weight (2 days at room temperature) were measured. In addition, seed germination (500 seeds per plant for each biotype) was estimated for seeds generated in both 2014 and 2015. The reduction in plant fitness associated with glyphosate resistance was estimated as: [1-(number of resistant seeds/number of susceptible seeds) × 100] (Yanniccari et al., 2016).

### Statistical Analysis

Dose-response and EPSPS enzyme activity data were subjected to non-linear regression analysis using a log-logistic equation: Y = c+{(d-c)/[1+(x/g)b]} (Ritz et al., 2015); where Y is the percentage of fresh weight, survival and/or EPSPS-inhibiting with respect to the control, c and d are the parameters corresponding to the lower and upper asymptotes, b is the slope of the curve at the inflection point, g is the herbicide rate at the inflection point (i.e., GR_50_, LD_50_ or I_50_), and × (independent variable) is the glyphosate dose. Using this equation, the amount of glyphosate needed to reduce the fresh weight (GR_50_), mortality (LD_50_), and to inhibit EPSPS activity (I_50_) by 50% of each biotype were calculated. Regression analyses were conducted using the *drc* package with program R version 3.2.5 (R Core Team, 2015), and the data were plotted using SigmaPlot 11.0 (Systat Software, Inc., United States). Resistance indices (RI = R/S) were calculated as the ratio of R to- S GR_50_, LD_50,_ or I_50_.

An analysis of variance (ANOVA) was conducted to test for differences between R- and S-biotypes with respect to the spray retention assay, accumulation of shikimate, glyphosate metabolite levels, ^14^C-glyphosate uptake and translocation, and basal enzyme activity, as well as plant growth and fecundity (fitness penalty). Differences between means were separated using the Tukey HSD test at *P* < 0.05. Model assumptions of normal distribution of errors and homogeneous variance were graphically inspected. All ANOVAs were conducted using Statistix (version. 9.0) (Analytical Software, United States) software.

## Results

### Identification of *Lolium* Weed Species Using Molecular Markers

**FIGURE 1:**
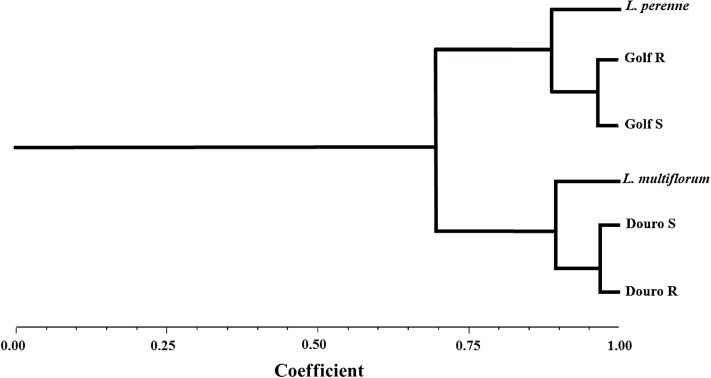
Dendogram of the genetic similarities among *Lolium* species after UPGMA analysis performed with AFLP marker data.

In the UPGMA (Unweighted Pair Group Method with Arithmetic Mean) dendogram, two main clusters converged at the 70% similarity level. The first cluster comprised a *L. perenne* control with R- and S-Golf biotypes. The second cluster was formed by *L. multiflorum* with R- and S-Douro biotypes. According to these results, the *Lolium* species used in this study clearly corresponded to *L. perenne* and *L. multiflorum* (**Figure 1**). Genetic characterization of the two species was necessary because *Lolium* species are obligate outcrossers; Salas et al. (2012) reported genetic variation for response to glyphosate from a collection of individual plants in the same location.

### Whole Plant Dose-Response

**FIGURE 2:**
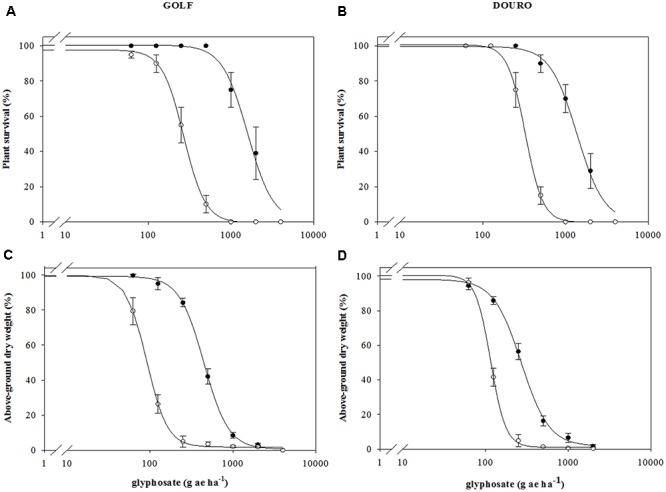
Mean glyphosate dose-response on plant survival **(A,B)** and above-ground dry weight **(C,D)** expressed as percentage of untreated control of glyphosate-resistant (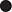) and -susceptible (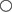) Golf and Douro biotypes. Values represent mean (*n* = 9) and vertical bars represent ± standard errors.

Survival (LD_50_) between R- and S-biotypes of Golf and Douro was different in the presence of glyphosate. Meanwhile the plants of the S-biotypes for each species died at 1000 g ha^-1^, but 70 and 80% of plants of the R-biotypes survived (**Figures 2A,B**). R-Golf exhibited 5.9-fold resistance (RI; resistance index) relative to S-Golf. Additionally, the R-Douro biotype showed 4.2-fold resistance to glyphosate relative to S-Douro (**Table 1**). Accumulation of plant biomass in the presence of glyphosate was also different between R- and S-biotypes of each species. Although mortality differences for R- and S-biotypes of both Golf and Douro were exhibited at 2000 g ha^-1^, dry weights were similar (**Figures 2C,D**). The RI for R-Golf and R-Douro was 4.9 and 2.3, respectively, in comparison to their respective S biotype (**Table 1**). Differences in both biomass accumulation and plant mortality confirm glyphosate resistance in R-Golf and R-Douro. The extent of resistance also suggests the potential underlying mechanism.

**Table 1:** Mortality (LD_50_), dry weight reduction (GR_50_), shikimic acid accumulation and inhibition of EPSPS enzyme activity (I_50_) by 50% in glyphosate-resistant (R) and -susceptible (S) Golf (*Lolium perenne*) and Douro (*L. multiflorum*) biotypes.

Biotype		Mortality reduction^a^			Dry weight reduction^a^			Shikimic acid^b^	EPSPS activity^c^		
		LD_50_	RI	*P*	GR_50_	RI	*P*		I_50_	RI	*P*
Golf	R	1590.6 ± 39.7	5.9	0.0001	443.8 ± 6.5	4.9	0.0001	36.5 ± 2.1	91.7 ± 6.9	28.7	0.0001
	S	267.0 ± 6.0			90.9 ± 1.8			214.9 ± 19.2	3.2 ± 0.3		
Douro	R	1375.2 ± 53.8	4.2	0.0001	271.5 ± 5.5	2.3	0.0001	45.4 ± 7.0	30.2 ± 4.3	10.8	0.0001
	S	326.5 ± 3.8			115.9 ± 1.4			193.6 ± 15.2	2.8 ± 0.2		

^a^*LD_50_ and GR_*50*_ values represent the glyphosate dose (g ae ha^-*1*^) needed to reduce mortality and dry weight, respectively by 50%. ±Standard error of the mean (*n* = 9). ^b^Shikimic acid accumulation (μg g^-*1*^ fresh weight) at 1000 μM glyphosate ± standard error of the mean (*n* = 9). ^c^I_*50*_ values represent the glyphosate dose (μM) needed to reduce inhibition of EPSPS activity by 50%. ±Standard error of the mean (*n* = 5). RI, Resistance indices (RI = R/S) calculated as the ratio of R to- S GR_*50*_, LD_*50*_ or I_*50*_ values. *P*, probability values*.

### Spray Retention

Leaf surface characteristics and plant architecture contribute to variability in the amount of herbicide solution retained by treated plants. The spray solution retained (mL per g dry weight) for the R- and S-Golf biotypes was 0.41 ± 0.08 and 0.48 ± 0.07 mL, respectively, which was significant (P = 0.004). For R- and S-Douro, spray retention was 0.32 ± 0.09 and 0.47 ± 0.06 mL of sprayed solution retained per g dry weight, respectively. These results were also significantly different between biotypes (*P* = 0.002). Reduced retention of 17 and 47% were evident for R-Golf and R-Douro, respectively.

### Shikimic Acid Accumulation

Shikimic acid levels in leaf segments of S-Golf and -Douro increased significantly by 24 h following exposure to 1000 μM glyphosate (**Table 1**). The R-Golf and -Douro biotypes accumulated 5.9- and 4.2-fold less shikimic acid, respectively than their respective S biotypes. Accumulation of shikimate in susceptible plants is a classic response to glyphosate; lack of shikimate accumulation in R-Golf and –Douro substantiates evidence for glyphosate resistance. The dynamics of shikimic acid accumulation were different between the S biotypes, showing that Golf was more sensitive to glyphosate than Douro. In both R biotypes, the accumulation of shikimic acid was limited and similar (**Table 1**).

### ^14^C-Glyphosate Uptake, Translocation, and Visualization

Total recovery of ^14^C-glyphosate in this research was 94.3, 95.1, 92.2, and 94.8% for R-Douro, S-Douro, R-Golf and S-Golf biotypes, respectively (data not shown). Little or no glyphosate was released by roots of treated plants from both Douro and Golf biotypes.

**FIGURE 3:**
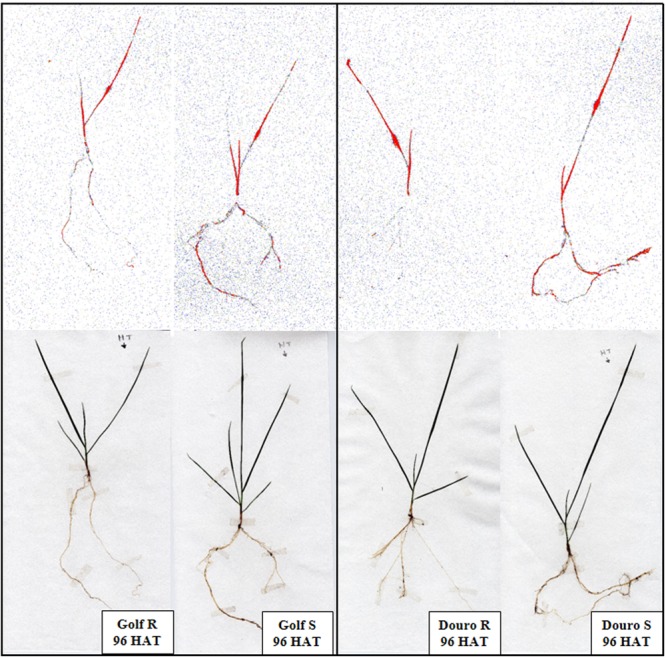
Visible movement of ^14^C-glyphosate in glyphosate-resistant and -susceptible Golf and Douro biotypes 96 h after an application to the treated leaf.

Uptake of ^14^C-glyphosate appeared to be bi-phasic, with uptake most rapid from 12 to 48 HAT, and slowing from 48 to 96 HAT. For Golf, glyphosate uptake through 24 HAT was similar for R- and S-biotypes (∼25% of maximum). From 24 through 96 HAT, uptake was more rapid in S- (82.7%) than R-Golf (56.6%). For Douro, ^14^C-glyphosate uptake was almost 2-fold higher for S- (50.7%) versus the R-biotype at 24 HAT. Differences in uptake continued through 96 HAT, with 91.2 and 50.7% total uptake for S- and R-Douro biotypes, respectively. In both R-biotypes, ^14^C-glyphosate levels in treated leaves (as a percentage of uptake) declined linearly from 12 to 96 HAT. However, the rate of movement out of the treated leaf for S-biotypes was greater compared to R-biotypes; 30.7 and 33.9% for Golf and Douro, respectively. Corresponding accumulation of ^14^C-glyphosate was measured in the remaining shoot tissue, although accumulation was 8.6 and 15.1% greater for S- compared to R-biotypes of Golf and Douro, respectively. Differences in ^14^C-glyphosate translocation to roots between S- and R-biotypes of each species were most noticeable beyond 48 HAT. S- compared to R-biotypes resulted in 2.4- and 2.8-fold higher levels of ^14^C-glyphosate in roots for Golf and Douro *Lolium*, respectively at 96 HAT (**Table 2**). The qualitative results obtained by phosphor imaging corroborated these differences in glyphosate distribution for S- versus R-biotypes of both species (**Figure 3**). These data reveal that restricting glyphosate uptake and translocation contributes to the survival of R-Golf and –Douro.

**Table 2:** Uptake and translocation of ^14^C-glyphosate from 12 to 96 h after treatment (HAT) in glyphosate-resistant (R) and -susceptible (S) Golf (*Lolium perenne*) and Douro (*L. multiflorum*) biotypes.

Biotype		HAT	Uptake (%)^a^	Translocation (%)^b^		
				Treated leaf	Rest of shoots	Roots
Golf	R	12	11.3 ± 2.8 G	95.9 ± 2.5 a	3.6 ± 0.7ef	0.5 ± 0.1f
		24	27.1 ± 3.9 F	89.2 ± 1.7 b	7.2 ± 1.6e	3.7 ± 0.9ef
		48	49.8 ± 3.4 E	76.0 ± 4.4 d	17.6 ± 2.5c	6.5 ± 1.4e
		72	54.3 ± 2.7 D	70.4 ± 3.4 e	18.2 @2.4c	11.4 ± 2.6d
		96	56.5 ± 1.3 D	59.8 ± 4.7 f	24.5 ± 4.7b	15.7 ± 3.1c
	S	12	10.3 ± 1.9 G	96.1 ± 3.1 a	2.7 ± 0.4f	1.2 ± 0.6f
		24	25.3 ± 2.3 F	81.2 ± 5.0 c	12.7 ± 4.0d	6.1 ± 2.1e
		48	66.5 ± 4.0 C	62.6 ± 1.7 f	29.7 ± 2.8a	7.7 ± 1.7de
		72	75.0 ± 1.7 B	38.9 ± 3.7 g	32.1 ± 4.0a	29.0 ± 4.7b
		96	82.7 ± 2.5 A	29.0 ± 2.8 h	33.1 ± 2.1a	37.9 ± 4.0a
Douro	R	12	8.5 ± 3.9 I	94.9 ± 2.4 a	2.8 ± 0.4f	2.3 ± 0.9ef
		24	16.9 ± 3.4 H	88.0 ± 1.4 b	9.0 ± 1.7e	3.0 ± 1.1ef
		48	40.0 ± 2.8 F	81.8 ± 2.9 c	10.2 ± 2.8e	7.9 ± 2.7cd
		72	45.3 ± 3.9 E	77.2 ± 3.6 d	15.0 ± 1.7d	8.1 ± 2.0cd
		96	50.7 ± 2.9 D	70.8 ± 4.3 e	19.1 ± 2.1c	10.1 ± 2.4bc
	S	12	16.8 ± 2.8 H	97.3 ± 1.4 a	1.5 ± 0.3f	1.2 ± 0.2f
		24	31.2 ± 3.2 G	80.8 ± 4.4 c	13.9 ± 1.9d	5.3 ± 1.2de
		48	73.6 ± 3.2 C	65.1 ± 3.9 f	22.0 ± 2.8c	13.0 ± 2.3b
		72	83.5 ± 2.0 B	41.0 ± 4.2 g	30.8 ± 3.1b	28.3 ± 3.8a
		96	91.2 ± 3.4 A	36.8 ± 2.6 h	34.2 ± 3.8a	29.0 ± 3.2a

*±Standard error of the mean (*n* = 6). Same letters in a column are not different at 95% by the Tukey’s test. ^a^Percent of applied label. ^b^Percent of uptake label*.

### Metabolism Study

For glyphosate absorbed into Golf and Douro biotypes, much of the herbicide was unaltered in plants by 96 HAT (>87%). The mean levels of AMPA ranged from 8.2 to 10.4% and were not different between R- and S-biotypes (**Table 3**). Glyoxylate levels ranged from 1.2 to 3.1%; R-Douro accumulated 65% more glyoxylate than S-Douro. Considering the small amount of glyoxylate formed, this difference is not likely biologically meaningful (**Table 3**). AMPA and glyoxylate are natural products of the degradation of glyphosate in some plants such as glyphosate-resistant soybean (Duke et al., 2003). The absence of differences in metabolite levels in R- and S-Golf and –Douro indicates this mechanism does not underlie resistance.

**Table 3:** Metabolism of glyphosate at 96 h after treatment in glyphosate-resistant (R) and -susceptible (S) Golf (*Lolium perenne*) and Douro (*L. multiflorum*) biotypes.

Biotypes		Glyphosate	AMPA	Glyoxylate
Golf	R	87.5 ± 3.2	10.4 ± 1.3	2.1 ± 0.8
	S	90.1 ± 5.3	8.7 ± 2.9	1.2 ± 0.2
	*P*	0.1652	0.2073	0.1263
Douro	R	88.7 ± 4.3	8.2 ± 3.7	3.1 ± 0.4 a
	S	89.0 ± 3.9	9.1 ± 3.2	1.9 ± 0.7 b
	*P*	0.2788	0.0786	0.0214

*±Standard error of the mean (*n* = 6). Different letters in a column are different at 95% by the Tukey’s test. AMPA, aminomethylphosphonic acid. Plants treated with 300 g ae ha^-1^ glyphosate at the 3–4 leaf growth stage*.

### EPSPS Enzyme Activity Assays

**FIGURE 4:**
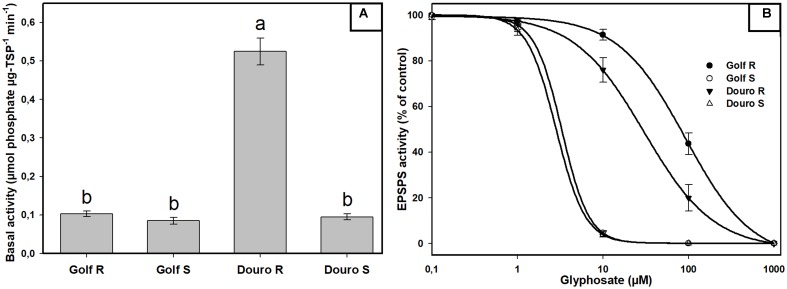
EPSPS enzyme expressed as a percentage of the untreated control in leaf extracts of glyphosate-resistant and -susceptible Golf and Douro biotypes. **(A)** Basal EPSPS activity where histograms represent treatment means and vertical bars ± standard error (*n* = 5). **(B)** EPSPS enzyme activity expressed as a percentage of the untreated control; vertical bars represent ± standard error (*n* = 5).

Examination of constitutive EPSPS activity in the absence of glyphosate (basal enzyme activity) revealed a striking difference between R- and S-Golf versus R- and S-Douro. Activity in R- and S-Golf was similar, with 0.097 and 0.085 μmol μg protein^-1^ min^-1^. However, basal enzyme activity of R-Douro was 5.2-fold higher compared to S-Douro (**Figure 4A**). Similar EPSPS activity in the absence of glyphosate for Golf biotypes is evidence that R-Golf does not exhibit overexpression of enzyme. However, an increase in basal EPSPS activity for R-Douro suggests EPSPS copy number may be elevated. Inhibition of EPSPS activity was achieved by challenging R- and S-biotypes of Golf and Douro with glyphosate (**Figure 4B**). The glyphosate rate necessary to inhibit EPSPS activity by 50% (I_50_) was 28.7-fold higher for R-versus S-Golf and 10.8-fold higher for R-versus S-Douro, respectively (**Table 1**). Elevation in the dose of glyphosate needed to attain an I_50_ value for R-versus S-Golf suggests reduced sensitivity of EPSPS.

### EPSPS Gene Sequence and Expression

**FIGURE 5:**
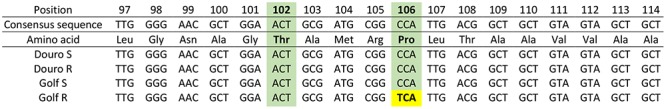
Partial nucleotide sequence of 5-enolpyruvlshikimate-3-phosphate synthase (EPSPS) DNA isolated from the glyphosate-resistant (R) and –susceptible (S) Golf and Douro biotypes.

The amplified fragment of the EPSPS gene included Thr-102 and Pro-106 positions, which frequently have been associated with conferring glyphosate resistance. In R- compared to S-Golf, a single codon change (from CCA to TCA) resulted in a conversion of the amino acid Pro to Ser at position 106. However, no nucleotide changes were observed for R-versus S-Douro. No additional codon changes in the Thr-102 position or others positions were observed for R-biotypes of either *Lolium* species (**Figure 5**). Clearly, a mutation in the EPSPS gene contributes to glyphosate resistance in R-Golf but not R-Douro.

**FIGURE 6:**
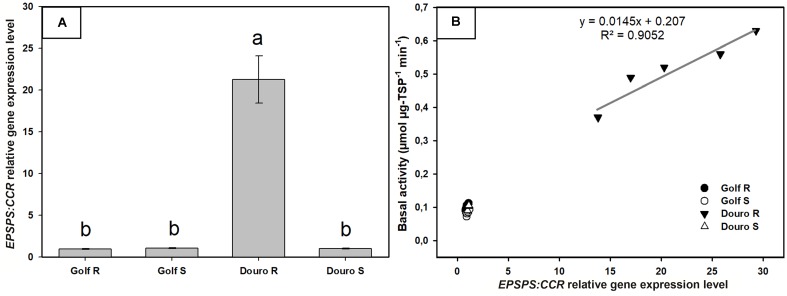
EPSPS gene expression relative to cinnamoyl-CoA reductase (*CCR*) gene in glyphosate-resistant and -susceptible Golf and Douro biotypes. **(A)** EPSPS expression level for glyphosate-susceptible and -resistant plants of Golf and Douro biotypes. Histograms represent the treatment means and vertical bars ± standard error (*n* = 6). **(B)** Correlation between the EPSPS expression and EPSPS basal activity.

The EPSPS gene expression, relative to the *CCR* gene quantified from cDNA in *Lolium* sp. biotypes, showed no differences between R-Golf, S-Golf and S-Douro ranging from 0.95 to 1.06. However, the EPSPS gene expression of the R-Douro biotype ranged from 13.8 to 29.2-fold (average of 21.2-fold) higher compared to the other *Lolium* biotypes. The EPSPS expression level of these biotypes was positively correlated with the EPSPS basal activity (**Figure 6**).

### Fitness Assessment

**FIGURE 7:**
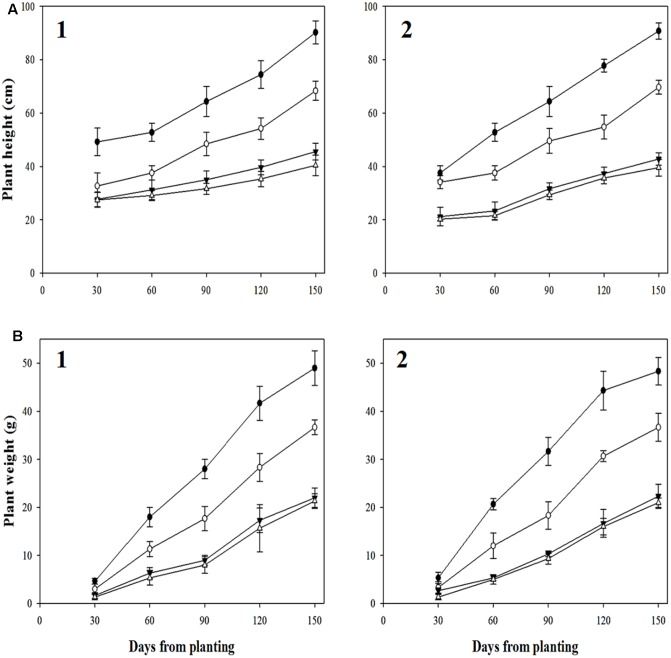
Plant height **(A)** and weight **(B)** of glyphosate-resistant Golf (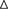) and Douro (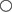) as well as glyphosate-susceptible Golf (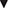) and Douro (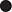) biotypes between 30 and 150 days after planting in 2014 (1) and 2015 (2). Values represent mean (*n* = 9) and vertical bars represent ± standard errors.

**FIGURE 8:**
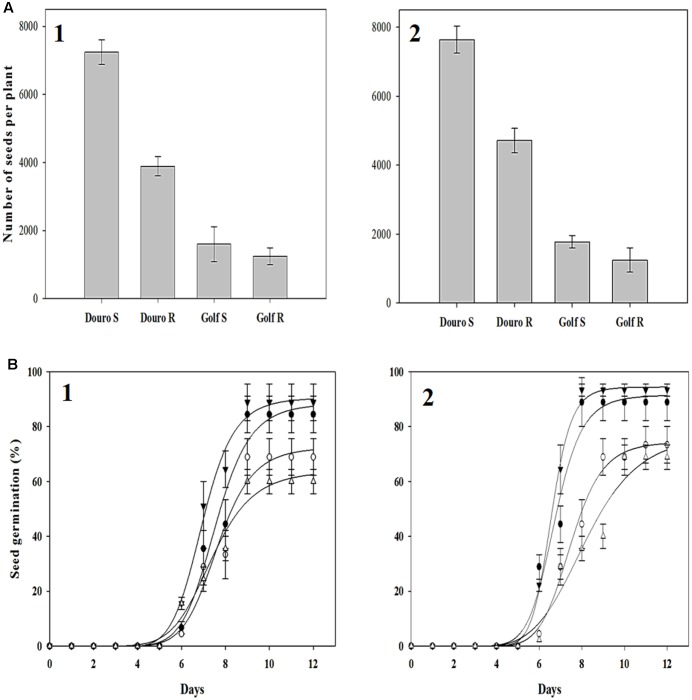
Harvested seed **(A)** and seed germination **(B)** from glyphosate-resistant Golf (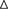) and Douro (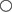) as well as glyphosate-susceptible Golf (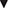) and Douro (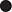) biotypes following seed harvest in 2014 (1) and 2015 (2). Harvested seed values represent mean (*n* = 9) and vertical bars represent ± standard errors. Germination values represent the mean (*n* = 500) at 12 days after seeding. Vertical bars represent ± standard errors.

Distinct differences between R- and S-biotypes of each species were observed for a number of vegetative and reproductive parameters (**Figures 7**, **8**). For Golf, the ANOVA (**Table 4**) comparing the vegetative parameters plant height and weight were significant (*P* < 0.05), but numerically close in value. However, the ANOVA comparing plant height and weight variables for the Douro biotypes were highly significant and numerically distinct. At 30 DAP, seedlings of S- versus R-Douro were 17.3 and 4.5% taller in 2014 and 2015, respectively. Plant height differences increased for the duration of the experiment, with S-Douro 32.1 and 30.3% taller than R-Douro at 150 DAP in 2014 and 2015, respectively. For Golf, plant height for R- and S-Golf was similar at 30 DAP, however, S-plants were 12.6 and 8.1% taller than R-plants 150 DAP in 2014 and 2015, respectively. Golf biotypes were shorter in height than Douro biotypes, likely a reflection of Golf being used in the turf industry. Plant biomass differences between R- and S-biotypes of both species followed differences in plant height (**Figure 7**). Over time, the biomass of S-Douro increased faster than that of R-Douro. At 150 DAP, biomass of S- versus R-Douro was 32 and 36.1% greater in 2014 and 2015, respectively. For Golf, biomass was similar for S- and R-biotypes throughout the duration of the experiment. By 150 DAP, biomass of S- versus R-Golf was 3.3 and 6.2% greater in 2014 and 2015, respectively (**Figure 7**). Biomass results were similar for 2014 and 2015. Growth parameters for Golf were similar over years (biotype^∗^year interaction); significance in the biotype^∗^year interaction for plant height of Douro indicate some variability between biotypes for 2014 compared to 2015 (**Table 4**).

**Table 4:** Probability values (*P*) to assess fitness of glyphosate-resistant and -susceptible Golf (*Lolium perenne*) and Douro (*L. multiflorum*) biotypes.

	Plant height	Plant weight	Seed number	Germination
**Golf**				
Biotype	*P* < 0.0001	P = 0.0337	P = 0.0614	P < 0.0001
Year	*P* < 0.0001	*P* = 0.6394	*P* = 0.1167	*P* < 0.0001
DAP	*P* < 0.0001	*P* < 0.0001		
Biotype^∗^Year	*P* = 0.1970	*P* = 1.000	*P* = 0.1193	*P* < 0.0001
Biotype^∗^DAP	*P* = 0.0658	*P* = 0.9968		
Year^∗^DAP	*P* < 0.0001	*P* = 0.6364		
Biotype^∗^Year^∗^DAP	*P* = 0.6804	*P* = 0.9361		
**Douro**				
Biotype	*P* < 0.0001	*P* < 0.0001	*P* < 0.0001	*P* < 0.0001
Year	*P* = 0.548	*P* = 0.47	*P* = 0.0024	*P* < 0.0001
DAP	*P* < 0.0001	*P* < 0.0001		
Biotype^∗^Year	*P* = 0.0288	*P* = 0.8504	*P* = 0.2028	*P* < 0.0001
Biotype^∗^DAP	*P* < 0.0001	*P* = 0.0002		
Year^∗^DAP	*P* = 0.0008	*P* = 0.5427		
Biotype ^∗^Year^∗^DAP	*P* < 0.0001	*P* = 0.7349		

*Variables included biotype, year (2014 or 2015), days after planting (DAP; 30, 60, 90, 120, and 150). Plant characteristics included plant height and biomass, seed production, and germination of harvested seed*.

Greater plant height and biomass for S- compared to R-biotypes of Douro impacted seed production. For Douro, mean seed production of S- versus R-biotypes was 3,355 and 2,926 seeds higher in 2014 and 2015, respectively. S-Golf produced 351 and 528 more seeds than the R-plants in 2014 and 2015, respectively. Seed germination of Golf and Douro biotypes ranged from 61 to 87% in 2014 and 66 to 92% in 2015. Initial germination was observed 4 days after seeding, and reached an optimum 12 days after seeding. For both species, germinability of the S-biotype was higher compared to the R-biotype. In 2014 and 2015, germination of S- versus R-Golf was 28.8 and 24.4% higher, respectively. Small differences in germinability were also observed for Douro. Seed germination for S- versus R-Douro was 15.5 and 20.0% higher in 2014 and 2015, respectively (**Figure 8**).

## Discussion

In this research, characterization of both NTSR and TSR mechanisms were explored, reflecting the most frequently expressed mechanisms in *Lolium* species. First, the *Lolium* species assayed by AFPL-markers were identified as *L. perenne* for the Golf biotypes and *L. multiflorum* for the Douro biotypes, revealing molecular-based relationships between three basic entities. The use of AFPL-markers allowed easy identification of species, but was not able to detect resistance to glyphosate between *Lolium* populations. AFLPs can produce biased results due to fragment-size homoplasy, caused by the lack of homology of fragments (Caballero et al., 2008; Chandi et al., 2013). Golf and Douro biotypes exhibit a high level of genetic similarity, showing minimal differences between R- and S-biotypes within each species (>0.95).

The occurrence of glyphosate resistance in *Lolium* spp. is not novel. Between 1996 and 2017, 14 countries documented R *Lolium* (Heap, 2017), the most occurring in *L. rigidum* and *L. multiflorum*. Levels of resistance in R-Golf (5.9) and R-Douro (4.2) (**Table 1**) were similar to that reported for other glyphosate-resistant biotypes (Perez-Jones et al., 2007; Jasieniuk et al., 2008; González-Torralva et al., 2012). Preston et al. (2009) summarized that R/S ratios for *L. multiflorum* biotypes with reduced translocation as the basis for resistance varied from 3 to 9. However, amino substitutions at Pro-106 to serine or alanine in 5 different biotypes resulted in R/S ratios from 5 to 15. In Argentina, *L. perenne* exhibited 10.8-fold resistance to glyphosate (Yanniccari et al., 2012b), with the mechanism later stated to be overexpression of EPSPS (Yanniccari et al., 2016).

Differences in retention of glyphosate on treated leaves has not been characterized as a resistance mechanism in selected weeds. The contact angle for R-versus S-biotypes of *L. multiflorum* was up to 30 degrees lower, resulting in 35% less glyphosate retained on leaves (Michitte et al., 2007). However, R-biotypes of *L. multiflorum* in Spain did not exhibit unique morphology compared to S-biotypes, and herbicide retention was similar (González-Torralva et al., 2012). A reduction in the amount of glyphosate retained on Golf and Douro plants would not preclude the herbicide’s physiological effects. Because biotype differences in plant mortality and biomass accumulation ranged from 230 to 590%, small differences in retention could not alone underlie R-Golf and –Douro resistance.

Shikimic acid accumulation in both S-Golf and -Douro indicated herbicide interaction with the target enzyme (EPSPS), but accumulation of shikimate was distinctively higher in S plants (**Table 1**). Inhibition of EPSPS concomitantly results in accumulation of shikimate in glyphosate sensitive plants (Maeda and Dudareva, 2012; Sammons and Gaines, 2014), suggesting limited interaction of glyphosate with EPSPS of R plants. Higher accumulation of shikimic acid in S- versus R-biotypes was similar to other *Lolium* populations (Michitte et al., 2007; Jasieniuk et al., 2008; Nandula et al., 2008), where shikimic acid levels 2- to 6-times higher in S- versus R-plants were observed. An S-biotype of *L. multiflorum* in Spain accumulated 7.3-fold more shikimic acid compared to the R-biotype (González-Torralva et al., 2012). For S- versus R-biotypes of *L. perenne* (Golf), also from Spain, the differences in accumulation of shikimic acid were less pronounced. S- versus R-plants of *L. perenne* reached levels approximately 3-fold higher by 72 h after glyphosate application (Yanniccari et al., 2012a). Therefore, limited increases in shikimic acid content in R-biotypes is an indicator of reduced sensitivity to glyphosate, but does not indicate the mechanism underlying resistance.

Reduced uptake and/or translocation is an underlying mechanism of glyphosate resistance and has been documented in a number of *Lolium* species (González-Torralva et al., 2012; Ghanizadeh et al., 2015; Salas et al., 2015). In *L. multiflorum* from Chile (Michitte et al., 2007) and Mississippi (Nandula et al., 2008), the limited uptake in R-biotypes corresponded with reduced movement out of labeled tissue or accumulation in tips of treated leaves. Greater translocation of ^14^C-glyphosate in S- versus R-plants of *L. perenne* resulted in accumulation in pseudostem regions (Ghanizadeh et al., 2015) or roots (Lorraine-Colwill et al., 2003; González-Torralva et al., 2012). Uptake of glyphosate across leaf cuticles is facilitated by a carrier-mediated process (Sterling, 1994), with maximum absorption into plants reached within 74 HAT (Li et al., 2005). Reduced uptake in R-Golf and R-Douro may be related to changes in cuticular properties, and be independent from mechanisms contributing to restricted movement of glyphosate (**Table 2**). Reduced uptake into R *L. multiflorum* from Chile was due to cuticular properties on the abaxial leaf surface (Michitte et al., 2007).

R plants of the Douro and Golf biotypes showed reduced glyphosate translocation (**Table 2**). The reduction in translocation to active and sensitive sites, such as root and shoot meristems, has a negative impact on glyphosate efficacy (Preston and Wakelin, 2008), contributing to the loss of herbicide sensitivity in R plants. The physiological mechanism that reduces glyphosate translocation in R-versus S-plants is not fully known (Lorraine-Colwill et al., 2003; Gherekhloo et al., 2017). However, it has been suggested that there is an unknown barrier in the phloem system or in the mesophyll cells (Yanniccari et al., 2012a). This barrier may alter the subcellular accumulation of glyphosate at the point where the herbicide is translocated (Kleinman and Rubin, 2017). The R plants of the Douro and Golf biotypes retained the herbicide applied mainly on the treated leaves. Similar translocation patterns were reported in R- and S-biotypes of *L. rigidum*, where 89 and 58%, respectively of applied ^14^C-glyphosate was retained in the treated leaves at 96 HAT (Fernández-Moreno et al., 2017b). In our research, NTSR mechanisms involving restricted uptake and translocation of glyphosate contribute to the resistance exhibited in R-biotypes of both *L. multiflorum* (Douro) and *L. perenne* (Golf).

Glyphosate degradation to the major metabolites AMPA and glyoxylate has been investigated in *Lolium* (Salas et al., 2012; González-Torralva et al., 2012; Fernandez et al., 2015) and other species (Feng et al., 2004; Cruz-Hipolito et al., 2011; Fernández-Moreno et al., 2016), and is low compared to metabolism in glyphosate-resistant crop plants (Duke, 2011). Lorraine-Colwill et al. (2003) and González-Torralva et al. (2012) in *L. multiflorum*, and Fernández-Moreno et al. (2017b) in *L. rigidum* concluded that metabolism does not contribute to resistance. With greater than 85% of absorbed glyphosate remaining unaltered both in S- as well as R- Douro and Golf biotypes (*L. multiflorum* and *L. perenne*, respectively) (**Table 3**), metabolism cannot explain the level of glyphosate resistance observed.

Elevated basal activity of EPSPS has been reported in R biotypes of *Lolium* species (Salas et al., 2012; Fernández-Moreno et al., 2016). The R-Douro biotype also exhibited higher basal activity (**Figure 4**). Differences in basal activity are explained by duplication of EPSPS gene copy number (Salas et al., 2012; Gherekhloo et al., 2017). Gene amplification allows plants to carry on normal metabolic activities in the presence of once lethal concentrations of glyphosate (Powles, 2010); significantly higher doses of glyphosate are necessary for complete inhibition of EPSPS in R plants. This explains why the R-Douro biotype required greater amounts of glyphosate to inhibit EPSPS activity by 50% (**Figure 4**). There is generally a positive correlation between EPSPS gene copy numbers and EPSPS transcription (Gaines et al., 2013; Salas et al., 2015). Our results suggest that R-Douro had a ratio of ± 21-fold more EPSPS gene copy number than S-Douro. Higher basal EPSPS activity associated with additional EPSPS gene copies was reported in R-plants of *L. perenne* (Salas et al., 2012, 2015), but our results revealed this mechanism for the first time in *L. multiflorum.*

Nucleotide substitution resulting in amino acid changes in the EPSPS gene have frequently conferred glyphosate-resistance in biotypes of *Lolium* (Perez-Jones et al., 2007; Jasieniuk et al., 2008; Ghanizadeh et al., 2015; Fernandez et al., 2015). In this research, R-Golf exhibited a 106-Pro to –Ser substitution, altering the binding of glyphosate to the target site (**Figure 5**). Sammons and Gaines (2014) summarized that Pro-106-Ser mutation was reported in *L. multiflorum* (Perez-Jones et al., 2007; Jasieniuk et al., 2008; González-Torralva et al., 2012), *L. rigidum* (Simarmata and Penner, 2008; Bostaman et al., 2012; Fernandez et al., 2015) and *L. perenne* (Ghanizadeh et al., 2015). The 106-Pro-Thr (Wakelin and Preston, 2006; Bostaman et al., 2012), Pro-106-Leu (Kaundun et al., 2011), and Pro-106-Ala (Yu et al., 2007) mutations were identified in *L. rigidum*. Variable amino acid substitutions impact the efficiency of glyphosate, resulting in variable levels of resistance (Preston et al., 2009).

As *Lolium* are an obligatory outcrossing species (Terrell, 1968), it is not unexpected that R *Lolium* populations exhibiting different mechanisms could hybridize, resulting in a progeny expressing more than one resistance mechanism. Resistant *L*. *multiflorum* populations from Oregon (Perez-Jones et al., 2007) and Spain (González-Torralva et al., 2012) showed altered translocation and a Pro-106-Ser mutation underlying resistance. Two mechanisms were also expressed in R *L. perenne* from New Zealand (Ghanizadeh et al., 2015). In this research, both R biotypes exhibit two resistance mechanisms. R-Golf expressed both reduced uptake and translocation (NTSR) as well as a 106-Pro-Ser mutation. R-Douro also demonstrates reduced uptake and translocation, but also overexpresses EPSPS. Recently, *E. indica* from Mexico was found to exhibit two TSR mechanisms (Gherekhloo et al., 2017).

Expression of glyphosate resistance in weed species via one or multiple mechanisms may impact the fitness of resistant biotypes. Cumulative germination of R goosegrass (*E. indica* Gaertn.) in Malaysia was higher compared to an S-population collected 2 km away (Ismail et al., 2002). The life cycle of R-glyphosate goosegrass may be shorter and integrated management techniques could reduce the incidence of R plants in the soil seed bank (Ismail et al., 2002). *L. rigidum* phenotypes from a single population did not differ in vegetative growth or competitive ability with wheat (*Triticum aestivum* L.), where R-versus S-plants generated 28% greater seed yield per plant, although seed number per plant was 7.5% less (Pedersen et al., 2007). R versus S *L*. *perenne* were shorter with lower shoot biomass and reduced leaf area, resulting in 40% fewer seeds in R plants (Yanniccari et al., 2016). Leaf areas of R and S *L. multiflorum* and *L. perenne* were not compared, but if lower in R-versus S-biotypes, this could impact glyphosate retention.

Determination of the growth and reproductive fitness of R-plants depends upon minimizing genetic variation. Ideally, isogenic lines of each biotype should be used, but this is often not achievable. The genetic variability of *Lolium* is high, which complicates fitness assessments. High variability among accessions within different classes of herbicide-resistant *L. rigidum* precludes identification of distinct emergence and growth characteristics (Gill et al., 1996). Differences in growth and fecundity can result from environmental conditions as well as the genetic background, as revealed by the UPGMA analysis in this research (**Figure 1**). This suggests minimal differences for fitness characteristics between R- and S-biotypes within each species are likely the result of differences in sensitivity to glyphosate.

Resistance to glyphosate based upon altered uptake and translocation appears to exact some influence on the fitness of the R-biotypes. R- and S-populations of *L. rigidum* were similar in vegetative growth in the absence or presence of competing wheat, but R-plants produced 3–9% more seed when competing with wheat at densities of 200 plants m^-2^ or higher (Pedersen et al., 2007). Under field conditions in the absence of glyphosate, survival of R-plants declined by almost 50% after three generations (Preston et al., 2009). The R-versus S-biotype of *L. multiflorum* (Douro) exhibited differences in growth (plant height and biomass) as well as reproductive (seed production and germination) characteristics (**Table 4** and **Figures 7**, **8**). In the absence of glyphosate use, the agricultural success of R-Golf and R-Douro is unknown. Certainly the fitness cost for a number of growth and reproductive parameters would collectively place R biotypes of each species at a competitive disadvantage compared to S biotypes.

Gene amplification of EPSPS may also be costly to growth and fecundity of R-plants. Height, biomass, leaf area and seed production of R versus S *L. perenne* from Argentina was significantly reduced (Yanniccari et al., 2016). The continued use of glyphosate was responsible for dominance of R-plants in the weed population. The level of fitness cost associated with R *L. perenne* from Argentina (Yanniccari et al., 2016) was similar to that reported in this research, although the basis of resistance was not due to EPSPS amplification. However, this mechanism was identified in *L. multiflorum* (R-Douro).

Fitness studies in plants with molecular changes in the EPSPS gene underlying glyphosate resistance have not been determined to be an ecological cost in R-plants. In *E. indica* clones from an R population in Malaysia, the RR TIPS mutants (carrying the Thr-102-Ile and Pro-106-Ser mutations) showed a slight fitness cost, but were out-performed over time by Rr TIPS mutants, which may suffer little if any fitness cost (Yu et al., 2015). R-plants of this species exhibited up to 74% greater emergence than S-plants, although subsequent growth characteristics were not studied (Ismail et al., 2002). In this research, R-glyphosate *L. perenne* (Golf) exhibits small differences in plant height as well as biomass compared to the *S*-glyphosate (**Figure 7**). Reduction in cumulative germination of R-*E. indica* (Ismail et al., 2002), suggest that non-glyphosate based management could reduce the incidence of the R-*Lolium* biotypes.

A common theme contributing to selection for glyphosate resistant weed populations is continuous use of the same herbicide mode of action over an extended period of time. If R- and S-biotypes exhibit similar traits for growth and fecundity, eliminating the use of glyphosate will not favor a shift in the balance of the endemic weed population for the S-biotype. However, association of a fitness penalty with TSR or NTSR mechanisms provides an opportunity to exploit the particular penalty and reduce the frequency of the R biotype. Using cloned plants of genetically similar *L. multiflorum* and *L. perenne*, there does appear to be a moderate to severe fitness penalty on the growth and fecundity of R-plants. This research is the first study examining fitness penalties for R-biotypes of *L. perenne* and *L. multiflorum* expressing two mechanisms of resistance to glyphosate. Additional studies are necessary under field conditions to demonstrate that reduced fitness of glyphosate-resistant plants can translate into meaningful reductions in the incidence of R-populations.

## Author Contributions

PF-M, RAC, RS, and RDP designed all experiments performed and data analysis; and PF-M, RAC, RS, and RDP wrote the paper.

## Conflict of Interest Statement

The authors declare that the research was conducted in the absence of any commercial or financial relationships that could be construed as a potential conflict of interest.
